# Supplementary material for: Personal Network Inference Unveils Heterogeneous Immune Response Patterns to Viral Infection in Children with Acute Wheezing
Source: J Pers Med. 2021 Dec 3;11(12):1293. doi: 10.3390/jpm11121293 (PMC8706513; doi:10.3390/jpm11121293)
Supplement: Supplementary file 1 [file jpm-11-01293-s001.zip › jpm-1455769-supplementary.pdf]

Table S1. Annotations for the 646 genes in the signature set that distinguished case samples from controls and convalescence samples.

Probe ID	Gene ID	Gene Name	Entrez ID	Chromosome Location
102724827_at	NA	NA	NA	NA
104326052_at	NA	NA	NA	NA
102725191_at	NA	NA	NA	NA
100616314_at	NA	NA	NA	NA
19_at	*ABCA1*	ATP binding cassette subfamily A member 1	19	9q31.1
103_at	*ADAR*	adenosine deaminase RNA specific	103	1q21.3
126_at	*ADH1C*	alcohol dehydrogenase 1C (class I), gamma polypeptide	126	4q23
214_at	*ALCAM*	activated leukocyte cell adhesion molecule	214	3q13.11
218_at	*ALDH3A1*	aldehyde dehydrogenase 3 family member A1	218	17p11.2
224_at	*ALDH3A2*	aldehyde dehydrogenase 3 family member A2	224	17p11.2
240_at	*ALOX5*	arachidonate 5-lipoxygenase	240	10q11.21
271_at	*AMPD2*	adenosine monophosphate deaminase 2	271	1p13.3
308_at	*ANXA5*	annexin A5	308	4q27
351_at	*APP*	amyloid beta precursor protein	351	21q21.3
360_at	*AQP3*	aquaporin 3 (Gill blood group)	360	9p13.3
362_at	*AQP5*	aquaporin 5	362	12q13.12
366_at	*AQP9*	aquaporin 9	366	15q21.3
383_at	*ARG1*	arginase 1	383	6q23.2
384_at	*ARG2*	arginase 2	384	14q24.1
397_at	*ARHGDIB*	Rho GDP dissociation inhibitor beta	397	12p12.3
467_at	*ATF3*	activating transcription factor 3	467	1q32.3
476_at	*ATP1A1*	ATPase Na+/K+ transporting subunit alpha 1	476	1p13.1
516_at	*ATP5MC1*	ATP synthase membrane subunit c locus 1	516	17q21.32
539_at	*ATP5PO*	ATP synthase peripheral stalk subunit OSCP	539	21q22.11
563_at	*AZGP1*	alpha-2-glycoprotein 1, zinc-binding	563	7q22.1
604_at	*BCL6*	BCL6 transcription repressor	604	3q27.3
651_at	*BMP3*	bone morphogenetic protein 3	651	4q21.21
658_at	*BMPR1B*	bone morphogenetic protein receptor type 1B	658	4q22.3
683_at	*BST1*	bone marrow stromal cell antigen 1	683	4p15.32
684_at	*BST2*	bone marrow stromal cell antigen 2	684	19p13.11
688_at	*KLF5*	Kruppel like factor 5	688	13q22.1

710_at	*SERPING1*	serpin family G member 1	710	11q12.1
719_at	*C3AR1*	complement C3a receptor 1	719	12p13.31
726_at	*CAPN5*	calpain 5	726	11q13.5
777_at	*CACNA1E*	calcium voltage-gated channel subunit alpha1 E	777	1q25.3
834_at	*CASP1*	caspase 1	834	11q22.3
838_at	*CASP5*	caspase 5	838	11q22.3
847_at	*CAT*	catalase	847	11p13
864_at	*RUNX3*	RUNX family transcription factor 3	864	1p36.11
868_at	*CBLB*	Cbl proto-oncogene B	868	3q13.11
902_at	*CCNH*	cyclin H	902	5q14.3
944_at	*TNFSF8*	TNF superfamily member 8	944	9q32-q33.1
952_at	*CD38*	CD38 molecule	952	4p15.32
962_at	*CD48*	CD48 molecule	962	1q23.3
963_at	*CD53*	CD53 molecule	963	1p13.3
973_at	*CD79A*	CD79a molecule	973	19q13.2
1040_at	*CDS1*	CDP-diacylglycerol synthase 1	1040	4q21.23
1043_at	*CD52*	CD52 molecule	1043	1p36.11
1069_at	*CETN2*	centrin 2	1069	Xq28
1075_at	*CTSC*	cathepsin C	1075	11q14.2
1084_at	*CEACAM3*	CEA cell adhesion molecule 3	1084	19q13.2
1119_at	*CHKA*	choline kinase alpha	1119	11q13.2
1130_at	*LYST*	lysosomal trafficking regulator	1130	1q42.3
1232_at	*CCR3*	C-C motif chemokine receptor 3	1232	3p21.31
1316_at	*KLF6*	Kruppel like factor 6	1316	10p15.2
1318_at	*SLC31A2*	solute carrier family 31 member 2	1318	9q32
1351_at	*COX8A*	cytochrome c oxidase subunit 8A	1351	11q13.1
1378_at	*CR1*	complement C3b/C4b receptor 1 (Knops blood group)	1378	1q32.2
1390_at	*CREM*	cAMP responsive element modulator	1390	10p11.21
1396_at	*CRIP1*	cysteine rich protein 1	1396	14q32.33
1432_at	*MAPK14*	mitogen-activated protein kinase 14	1432	6p21.31
1435_at	*CSF1*	colony stimulating factor 1	1435	1p13.3
1462_at	*VCAN*	versican	1462	5q14.2-q14.3
1517_at	*CTSLP2*	cathepsin L pseudogene 2	1517	10q11.22
1540_at	*CYLD*	CYLD lysine 63 deubiquitinase	1540	16q12.1
1580_at	*CYP4B1*	cytochrome P450 family 4 subfamily B member 1	1580	1p33
1673_at	*DEFB4A*	defensin beta 4A	1673	8p23.1
1728_at	*NQO1*	NAD(P)H quinone dehydrogenase 1	1728	16q22.1
1739_at	*DLG1*	discs large MAGUK scaffold protein 1	1739	3q29
1806_at	*DPYD*	dihydropyrimidine dehydrogenase	1806	1p21.3

1836_at	*SLC26A2*	solute carrier family 26 member 2	1836	5q32
1844_at	*DUSP2*	dual specificity phosphatase 2	1844	2q11.2
1880_at	*GPR183*	G protein-coupled receptor 183	1880	13q32.3
1889_at	*ECE1*	endothelin converting enzyme 1	1889	1p36.12
1890_at	*TYMP*	thymidine phosphorylase	1890	22q13.33
1937_at	*EEF1G*	eukaryotic translation elongation factor 1 gamma	1937	11q12.3
2001_at	*ELF5*	E74 like ETS transcription factor 5	2001	11p13
2013_at	*EMP2*	epithelial membrane protein 2	2013	16p13.13
2040_at	*STOM*	stomatin	2040	9q33.2
2070_at	*EYA4*	EYA transcriptional coactivator and phosphatase 4	2070	6q23.2
2153_at	*F5*	coagulation factor V	2153	1q24.2
2180_at	*ACSL1*	acyl-CoA synthetase long chain family member 1	2180	4q35.1
2181_at	*ACSL3*	acyl-CoA synthetase long chain family member 3	2181	2q36.1
2182_at	*ACSL4*	acyl-CoA synthetase long chain family member 4	2182	Xq23
2191_at	*FAP*	fibroblast activation protein alpha	2191	2q24.2
2195_at	*FAT1*	FAT atypical cadherin 1	2195	4q35.2
2207_at	*FCER1G*	Fc fragment of IgE receptor Ig	2207	1q23.3
2268_at	*FGR*	FGR proto-oncogene, Src family tyrosine kinase	2268	1p35.3
2289_at	*FKBP5*	FKBP prolyl isomerase 5	2289	6p21.31
2319_at	*FLOT2*	flotillin 2	2319	17q11.2
2354_at	*FOSB*	FosB proto-oncogene, AP-1 transcription factor subunit	2354	19q13.32
2358_at	*FPR2*	formyl peptide receptor 2	2358	19q13.41
2537_at	*IFI6*	interferon alpha inducible protein 6	2537	1p35.3
2629_at	*GBA*	glucosylceramidase beta	2629	1q22
2643_at	*GCH1*	GTP cyclohydrolase 1	2643	14q22.2
2729_at	*GCLC*	glutamate-cysteine ligase catalytic subunit	2729	6p12.1
2799_at	*GNS*	glucosamine (N-acetyl)-6-sulfatase	2799	12q14.3
2877_at	*GPX2*	glutathione peroxidase 2	2877	14q23.3
2879_at	*GPX4*	glutathione peroxidase 4	2879	19p13.3
2938_at	*GSTA1*	glutathione S-transferase alpha 1	2938	6p12.2
2939_at	*GSTA2*	glutathione S-transferase alpha 2	2939	6p12.2
2992_at	*GYG1*	glycogenin 1	2992	3q24
3005_at	*H1-0*	H1.0 linker histone	3005	22q13.1
3101_at	*HK3*	hexokinase 3	3101	5q35.2

3108_at	*HLA-DMA*	major histocompatibility complex, class II, DM alpha	3108	6p21.32
3113_at	*HLA-DPA1*	major histocompatibility complex, class II, DP alpha 1	3113	6p21.32
3115_at	*HLA-DPB1*	major histocompatibility complex, class II, DP beta 1	3115	6p21.32
3122_at	*HLA-DRA*	major histocompatibility complex, class II, DR alpha	3122	6p21.32
3127_at	*HLA-DRB5*	major histocompatibility complex, class II, DR beta 5	3127	6p21.32
3142_at	*HLX*	H2.0 like homeobox	3142	1q41
3148_at	*HMGB2*	high mobility group box 2	3148	4q34.1
3240_at	*HP*	haptoglobin	3240	16q22.2
3371_at	*TNC*	tenascin C	3371	9q33.1
3385_at	*ICAM3*	intercellular adhesion molecule 3	3385	19p13.2
3428_at	*IFI16*	interferon gamma inducible protein 16	3428	1q23.1
3430_at	*IFI35*	interferon induced protein 35	3430	17q21.31
3431_at	*SP110*	SP110 nuclear body protein	3431	2q37.1
3433_at	*IFIT2*	interferon induced protein with tetratricopeptide repeats 2	3433	10q23.31
3434_at	*IFIT1*	interferon induced protein with tetratricopeptide repeats 1	3434	10q23.31
3437_at	*IFIT3*	interferon induced protein with tetratricopeptide repeats 3	3437	10q23.31
3455_at	*IFNAR2*	interferon alpha and beta receptor subunit 2	3455	21q22.11
3458_at	*IFNG*	interferon gamma	3458	12q15
3512_at	*JCHAIN*	joining chain of multimeric IgA and IgM	3512	4q13.3
3554_at	*IL1R1*	interleukin 1 receptor type 1	3554	2q11.2-q12.1
3660_at	*IRF2*	interferon regulatory factor 2	3660	4q35.1
3665_at	*IRF7*	interferon regulatory factor 7	3665	11p15.5
3669_at	*ISG20*	interferon stimulated exonuclease gene 20	3669	15q26.1
3684_at	*ITGAM*	integrin subunit alpha M	3684	16p11.2
3753_at	*KCNE1*	potassium voltage-gated channel subfamily E regulatory subunit 1	3753	21q22.12
3759_at	*KCNJ2*	potassium inwardly rectifying channel subfamily J member 2	3759	17q24.3
3815_at	*KIT*	KIT proto-oncogene, receptor tyrosine kinase	3815	4q12
3837_at	*KPNB1*	karyopherin subunit beta 1	3837	17q21.32
3903_at	*LAIR1*	leukocyte associated immunoglobulin like receptor 1	3903	19q13.42

3934_at	*LCN2*	lipocalin 2	3934	9q34.11
3959_at	*LGALS3BP*	galectin 3 binding protein	3959	17q25.3
3985_at	*LIMK2*	LIM domain kinase 2	3985	22q12.2
4001_at	*LMNB1*	lamin B1	4001	5q23.2
4015_at	*LOX*	lysyl oxidase	4015	5q23.1
4033_at	*LRMP*	lymphoid restricted membrane protein	4033	12p12.1
4051_at	*CYP4F3*	cytochrome P450 family 4 subfamily F member 3	4051	19p13.12
4061_at	*LY6E*	lymphocyte antigen 6 family member E	4061	8q24.3
4128_at	*MAOA*	monoamine oxidase A	4128	Xp11.3
4210_at	*MEFV*	MEFV innate immuity regulator, pyrin	4210	16p13.3
4213_at	*MEIS3P1*	Meis homeobox 3 pseudogene 1	4213	17p12
4299_at	*AFF1*	AF4/FMR2 family member 1	4299	4q21.3-q22.1
4306_at	*NR3C2*	nuclear receptor subfamily 3 group C member 2	4306	4q31.23
4311_at	*MME*	membrane metalloendopeptidase	4311	3q25.2
4343_at	*MOV10*	Mov10 RISC complex RNA helicase	4343	1p13.2
4430_at	*MYO1B*	myosin IB	4430	2q32.3
4495_at	*MT1G*	metallothionein 1G	4495	16q13
4501_at	*MT1X*	metallothionein 1X	4501	16q13
4502_at	*MT2A*	metallothionein 2A	4502	16q13
4520_at	*MTF1*	metal regulatory transcription factor 1	4520	1p34.3
4599_at	*MX1*	MX dynamin like GTPase 1	4599	21q22.3
4600_at	*MX2*	MX dynamin like GTPase 2	4600	21q22.3
4642_at	*MYO1D*	myosin ID	4642	17q11.2
4646_at	*MYO6*	myosin VI	4646	6q14.1
4671_at	*NAIP*	NLR family apoptosis inhibitory protein	4671	5q13.2
4924_at	*NUCB1*	nucleobindin 1	4924	19q13.33
4929_at	*NR4A2*	nuclear receptor subfamily 4 group A member 2	4929	2q24.1
4938_at	*OAS1*	2'-5'-oligoadenylate synthetase 1	4938	12q24.13
4939_at	*OAS2*	2'-5'-oligoadenylate synthetase 2	4939	12q24.13
4940_at	*OAS3*	2'-5'-oligoadenylate synthetase 3	4940	12q24.13
5027_at	*P2RX7*	purinergic receptor P2X 7	5027	12q24.31
5058_at	*PAK1*	p21 (RAC1) activated kinase 1	5058	11q13.5-q14.1
5140_at	*PDE3B*	phosphodiesterase 3B	5140	11p15.2
5175_at	*PECAM1*	platelet and endothelial cell adhesion molecule 1	5175	17q23.3
5176_at	*SERPINF1*	serpin family F member 1	5176	17p13.3
5265_at	*SERPINA1*	serpin family A member 1	5265	14q32.13
5321_at	*PLA2G4A*	phospholipase A2 group IVA	5321	1q31.1

5349_at	*FXYD3*	FXYD domain containing ion transport regulator 3	5349	19q13.12
5359_at	*PLSCR1*	phospholipid scramblase 1	5359	3q24
5371_at	*PML*	promyelocytic leukemia	5371	15q24.1
5542_at	*PRB1*	proline rich protein BstNI subfamily 1 (gene/pseudogene)	5542	12p13.2
5563_at	*PRKAA2*	protein kinase AMP-activated catalytic subunit alpha 2	5563	1p32.2
5608_at	*MAP2K6*	mitogen-activated protein kinase kinase 6	5608	17q24.3
5610_at	*EIF2AK2*	eukaryotic translation initiation factor 2 alpha kinase 2	5610	2p22.2
5782_at	*PTPN12*	protein tyrosine phosphatase non-receptor type 12	5782	7q11.23
5783_at	*PTPN13*	protein tyrosine phosphatase non-receptor type 13	5783	4q21.3
5859_at	*QARS1*	glutaminyl-tRNA synthetase 1	5859	3p21.31
5899_at	*RALB*	RAS like proto-oncogene B	5899	2q14.2
5939_at	*RBMS2*	RNA binding motif single stranded interacting protein 2	5939	12q13.3
5966_at	*REL*	REL proto-oncogene, NF-kB subunit	5966	2p16.1
5991_at	*RFX3*	regulatory factor X3	5991	9p24.2
6023_at	*RMRP*	RNA component of mitochondrial RNA processing endoribonuclease	6023	9p13.3
6083_at	*SNORD21*	small nucleolar RNA, C/D box 21	6083	1p22.1
6125_at	*RPL5*	ribosomal protein L5	6125	1p22.1
6147_at	*RPL23A*	ribosomal protein L23a	6147	17q11.2
6155_at	*RPL27*	ribosomal protein L27	6155	17q21.31
6156_at	*RPL30*	ribosomal protein L30	6156	8q22.2
6165_at	*RPL35A*	ribosomal protein L35a	6165	3q29
6193_at	*RPS5*	ribosomal protein S5	6193	19q13.43
6205_at	*RPS11*	ribosomal protein S11	6205	19q13.33
6207_at	*RPS13*	ribosomal protein S13	6207	11p15.1
6223_at	*RPS19*	ribosomal protein S19	6223	19q13.2
6283_at	*S100A12*	S100 calcium binding protein A12	6283	1q21.3
6289_at	*SAA2*	serum amyloid A2	6289	11p15.1
6338_at	*SCNN1B*	sodium channel epithelial 1 beta subunit	6338	16p12.2
6342_at	*SCP2*	sterol carrier protein 2	6342	1p32.3
6355_at	*CCL8*	C-C motif chemokine ligand 8	6355	17q12
6373_at	*CXCL11*	C-X-C motif chemokine ligand 11	6373	4q21.1
6385_at	*SDC4*	syndecan 4	6385	20q13.12
6398_at	*SECTM1*	secreted and transmembrane 1	6398	17q25.3
6402_at	*SELL*	selectin L	6402	1q24.2

6446_at	*SGK1*	serum/glucocorticoid regulated kinase 1	6446	6q23.2
6480_at	*ST6GAL1*	ST6 beta-galactoside alpha-2,6-sialyltransferase 1	6480	3q27.3
6498_at	*SKIL*	SKI like proto-oncogene	6498	3q26.2
6503_at	*SLA*	Src like adaptor	6503	8q24.22
6507_at	*SLC1A3*	solute carrier family 1 member 3	6507	5p13.2
6541_at	*SLC7A1*	solute carrier family 7 member 1	6541	13q12.3
6546_at	*SLC8A1*	solute carrier family 8 member A1	6546	2p22.1
6556_at	*SLC11A1*	solute carrier family 11 member 1	6556	2q35
6565_at	*SLC15A2*	solute carrier family 15 member 2	6565	3q13.33
6583_at	*SLC22A4*	solute carrier family 22 member 4	6583	5q31.1
6594_at	*SMARCA1*	SWI/SNF related, matrix associated, actin dependent regulator of chromatin, subfamily a, member 1	6594	Xq25-q26.1
6614_at	*SIGLEC1*	sialic acid binding Ig like lectin 1	6614	20p13
6672_at	*SP100*	SP100 nuclear antigen	6672	2q37.1
6696_at	*SPP1*	secreted phosphoprotein 1	6696	4q22.1
6702_at	*SPRR2C*	small proline rich protein 2C (pseudogene)	6702	1q21.3
6705_at	*SPRR2F*	small proline rich protein 2F	6705	1q21.3
6707_at	*SPRR3*	small proline rich protein 3	6707	1q21.3
6732_at	*SRPK1*	SRSF protein kinase 1	6732	6p21.31
6737_at	*TRIM21*	tripartite motif containing 21	6737	11p15.4
6773_at	*STAT2*	signal transducer and activator of transcription 2	6773	12q13.3
6774_at	*STAT3*	signal transducer and activator of transcription 3	6774	17q21.2
6868_at	*ADAM17*	ADAM metallopeptidase domain 17	6868	2p25.1
6890_at	*TAP1*	transporter 1, ATP binding cassette subfamily B member	6890	6p21.32
6920_at	*TCEA3*	transcription elongation factor A3	6920	1p36.12
7033_at	*TFF3*	trefoil factor 3	7033	21q22.3
7057_at	*THBS1*	thrombospondin 1	7057	15q14
7076_at	*TIMP1*	TIMP metallopeptidase inhibitor 1	7076	Xp11.3
7096_at	*TLR1*	toll like receptor 1	7096	4p14
7097_at	*TLR2*	toll like receptor 2	7097	4q31.3
7099_at	*TLR4*	toll like receptor 4	7099	9q33.1
7100_at	*TLR5*	toll like receptor 5	7100	1q41
7113_at	*TMPRSS2*	transmembrane serine protease 2	7113	21q22.3
7114_at	*TMSB4X*	thymosin beta 4 X-linked	7114	Xp22.2
7130_at	*TNFAIP6*	TNF alpha induced protein 6	7130	2q23.3
7150_at	*TOP1*	DNA topoisomerase I	7150	20q12
7358_at	*UGDH*	UDP-glucose 6-dehydrogenase	7358	4p14

7431_at	*VIM*	vimentin	7431	10p13
7498_at	*XDH*	xanthine dehydrogenase	7498	2p23.1
7525_at	*YES1*	YES proto-oncogene 1, Src family tyrosine kinase	7525	18p11.32
7706_at	*TRIM25*	tripartite motif containing 25	7706	17q22
7832_at	*BTG2*	BTG anti-proliferation factor 2	7832	1q32.1
7850_at	*IL1R2*	interleukin 1 receptor type 2	7850	2q11.2
7940_at	*LST1*	leukocyte specific transcript 1	7940	6p21.33
7994_at	*KAT6A*	lysine acetyltransferase 6A	7994	8p11.21
8013_at	*NR4A3*	nuclear receptor subfamily 4 group A member 3	8013	9q31.1
8139_at	*GAN*	gigaxonin	8139	16q23.2
8291_at	*DYSF*	dysferlin	8291	2p13.2
8338_at	*H2AC20*	H2A clustered histone 20	8338	1q21.2
8342_at	*H2BC14*	H2B clustered histone 14	8342	6p22.1
8351_at	*H3C4*	H3 clustered histone 4	8351	6p22.2
8418_at	*CMAHP*	cytidine monophospho-N-acetylneuraminic acid hydroxylase, pseudogene	8418	6p22.3
8455_at	*ATRN*	attractin	8455	20p13
8519_at	*IFITM1*	interferon induced transmembrane protein 1	8519	11p15.5
8522_at	*GAS7*	growth arrest specific 7	8522	17p13.1
8530_at	*CST7*	cystatin F	8530	20p11.21
8553_at	*BHLHE40*	basic helix-loop-helix family member e40	8553	3p26.1
8611_at	*PLPP1*	phospholipid phosphatase 1	8611	5q11.2
8612_at	*PLPP2*	phospholipid phosphatase 2	8612	19p13.3
8638_at	*OASL*	2'-5'-oligoadenylate synthetase like	8638	12q24.31
8660_at	*IRS2*	insulin receptor substrate 2	8660	13q34
8667_at	*EIF3H*	eukaryotic translation initiation factor 3 subunit H	8667	8q23.3-q24.11
8754_at	*ADAM9*	ADAM metallopeptidase domain 9	8754	8p11.22
8775_at	*NAPA*	NSF attachment protein alpha	8775	19q13.32-q13.33
8807_at	*IL18RAP*	interleukin 18 receptor accessory protein	8807	2q12.1
8809_at	*IL18R1*	interleukin 18 receptor 1	8809	2q12.1
8842_at	*PROM1*	prominin 1	8842	4p15.32
8847_at	*DLEU2*	deleted in lymphocytic leukemia 2	8847	13q14.2
8876_at	*VNN1*	vanin 1	8876	6q23.2
8972_at	*MGAM*	maltase-glucoamylase	8972	7q34
8976_at	*WASL*	WASP like actin nucleation promoting factor	8976	7q31.32

9034_at	*CCRL2*	C-C motif chemokine receptor like 2	9034	3p21.31
9050_at	*PSTPIP2*	proline-serine-threonine phosphatase interacting protein 2	9050	c("18q21.1", "tdb7990")
9071_at	*CLDN10*	claudin 10	9071	13q32.1
9111_at	*NMI*	N-myc and STAT interactor	9111	2q23.3
9122_at	*SLC16A4*	solute carrier family 16 member 4	9122	1p13.3
9124_at	*PDLIM1*	PDZ and LIM domain 1	9124	10q23.33
9168_at	*TMSB10*	thymosin beta 10	9168	2p11.2
9182_at	*RASSF9*	Ras association domain family member 9	9182	12q21.31
9211_at	*LGI1*	leucine rich glioma inactivated 1	9211	10q23.33
9246_at	*UBE2L6*	ubiquitin conjugating enzyme E2 L6	9246	11q12.1
9304_at	*SNORD22*	small nucleolar RNA, C/D box 22	9304	11q12.3
9308_at	*CD83*	CD83 molecule	9308	6p23
9332_at	*CD163*	CD163 molecule	9332	12p13.31
9437_at	*NCR1*	natural cytotoxicity triggering receptor 1	9437	19q13.42
9447_at	*AIM2*	absent in melanoma 2	9447	1q23.1-q23.2
9592_at	*IER2*	immediate early response 2	9592	19p13.13
9620_at	*CELSR1*	cadherin EGF LAG seven-pass G-type receptor 1	9620	22q13.31
9636_at	*ISG15*	ISG15 ubiquitin like modifier	9636	1p36.33
9674_at	*KIAA0040*	KIAA0040	9674	1q25.1
9683_at	*N4BP1*	NEDD4 binding protein 1	9683	16q12.1
9839_at	*ZEB2*	zinc finger E-box binding homeobox 2	9839	2q22.3
9957_at	*HS3ST1*	heparan sulfate-glucosamine 3-sulfotransferase 1	9957	4p15.33
10092_at	*ARPC5*	actin related protein 2/3 complex subunit 5	10092	1q25.3
10100_at	*TSPAN2*	tetraspanin 2	10100	1p13.2
10209_at	*EIF1*	eukaryotic translation initiation factor 1	10209	17q21.2
10225_at	*CD96*	CD96 molecule	10225	3q13.13-q13.2
10232_at	*MSLN*	mesothelin	10232	16p13.3
10346_at	*TRIM22*	tripartite motif containing 22	10346	11p15.4
10379_at	*IRF9*	interferon regulatory factor 9	10379	14q12
10397_at	*NDRG1*	N-myc downstream regulated 1	10397	8q24.22
10404_at	*CPQ*	carboxypeptidase Q	10404	8q22.1
10410_at	*IFITM3*	interferon induced transmembrane protein 3	10410	11p15.5
10412_at	*NSA2*	NSA2 ribosome biogenesis factor	10412	5q13.3
10435_at	*CDC42EP2*	CDC42 effector protein 2	10435	11q13.1
10437_at	*IFI30*	IFI30 lysosomal thiol reductase	10437	19p13.11
10475_at	*TRIM38*	tripartite motif containing 38	10475	6p22.2
10497_at	*UNC13B*	unc-13 homolog B	10497	9p13.3
10512_at	*SEMA3C*	semaphorin 3C	10512	7q21.11

10561_at	*IFI44*	interferon induced protein 44	10561	1p31.1
10568_at	*SLC34A2*	solute carrier family 34 member 2	10568	4p15.2
10579_at	*TACC2*	transforming acidic coiled-coil containing protein 2	10579	10q26.13
10628_at	*TXNIP*	thioredoxin interacting protein	10628	1q21.1
10645_at	*CAMKK2*	calcium/calmodulin dependent protein kinase kinase 2	10645	12q24.31
10673_at	*TNFSF13B*	TNF superfamily member 13b	10673	13q33.3
10791_at	*VAMP5*	vesicle associated membrane protein 5	10791	2p11.2
10797_at	*MTHFD2*	methylenetetrahydrofolate dehydrogenase (NADP+ dependent) 2, methenyltetrahydrofolate cyclohydrolase	10797	2p13.1
10800_at	*CYSLTR1*	cysteinyl leukotriene receptor 1	10800	Xq21.1
10855_at	*HPSE*	heparanase	10855	4q21.23
10870_at	*HCST*	hematopoietic cell signal transducer	10870	19q13.12
10875_at	*FGL2*	fibrinogen like 2	10875	7q11.23
10915_at	*TCERG1*	transcription elongation regulator 1	10915	5q32
10964_at	*IFI44L*	interferon induced protein 44 like	10964	1p31.1
11025_at	*LILRB3*	leukocyte immunoglobulin like receptor B3	11025	19q13.42
11040_at	*PIM2*	Pim-2 proto-oncogene, serine/threonine kinase	11040	Xp11.23
11074_at	*TRIM31*	tripartite motif containing 31	11074	6p22.1
11098_at	*PRSS23*	serine protease 23	11098	11q14.2
11177_at	*BAZ1A*	bromodomain adjacent to zinc finger domain 1A	11177	14q13.1-q13.2
11224_at	*RPL35*	ribosomal protein L35	11224	9q33.3
11237_at	*RNF24*	ring finger protein 24	11237	20p13
11274_at	*USP18*	ubiquitin specific peptidase 18	11274	22q11.21
11275_at	*KLHL2*	kelch like family member 2	11275	4q32.3
22797_at	*TFEC*	transcription factor EC	22797	7q31.2
22861_at	*NLRP1*	NLR family pyrin domain containing 1	22861	17p13.2
22904_at	*SBNO2*	strawberry notch homolog 2	22904	19p13.3
22990_at	*PCNX1*	pecanex 1	22990	14q24.2
23001_at	*WDFY3*	WD repeat and FYVE domain containing 3	23001	4q21.23
23015_at	*GOLGA8A*	golgin A8 family member A	23015	15q14
23130_at	*ATG2A*	autophagy related 2A	23130	11q13.1
23150_at	*FRMD4B*	FERM domain containing 4B	23150	3p14.1
23200_at	*ATP11B*	ATPase phospholipid transporting 11B (putative)	23200	3q26.33
23228_at	*PLCL2*	phospholipase C like 2	23228	3p24.3
23325_at	*WASHC4*	WASH complex subunit 4	23325	12q23.3
23327_at	*NEDD4L*	NEDD4 like E3 ubiquitin protein ligase	23327	18q21.31

23406_at	*COTL1*	coactosin like F-actin binding protein 1	23406	16q24.1
23424_at	*TDRD7*	tudor domain containing 7	23424	9q22.33
23558_at	*WBP2*	WW domain binding protein 2	23558	17q25.1
23584_at	*VSIG2*	V-set and immunoglobulin domain containing 2	23584	11q24.2
23586_at	*DDX58*	DExD/H-box helicase 58	23586	9p21.1
23597_at	*ACOT9*	acyl-CoA thioesterase 9	23597	Xp22.11
23601_at	*CLEC5A*	C-type lectin domain containing 5A	23601	7q34
23643_at	*LY96*	lymphocyte antigen 96	23643	8q21.11
23765_at	*IL17RA*	interleukin 17 receptor A	23765	22q11.1
24138_at	*IFIT5*	interferon induced protein with tetratricopeptide repeats 5	24138	10q23.31
25824_at	*PRDX5*	peroxiredoxin 5	25824	11q13.1
25911_at	*DPCD*	deleted in primary ciliary dyskinesia homolog (mouse)	25911	10q24.32
25932_at	*CLIC4*	chloride intracellular channel 4	25932	1p36.11
25939_at	*SAMHD1*	SAM and HD domain containing deoxynucleoside triphosphate triphosphohydrolase 1	25939	20q11.23
26118_at	*WSB1*	WD repeat and SOCS box containing 1	26118	17q11.1
26154_at	*ABCA12*	ATP binding cassette subfamily A member 12	26154	2q35
26253_at	*CLEC4E*	C-type lectin domain family 4 member E	26253	12p13.31
26298_at	*EHF*	ETS homologous factor	26298	11p13
26471_at	*NUPR1*	nuclear protein 1, transcriptional regulator	26471	16p11.2
26777_at	*SNORA71A*	small nucleolar RNA, H/ACA box 71A	26777	20q11.23
26780_at	*SNORA68*	small nucleolar RNA, H/ACA box 68	26780	19p13.11
26781_at	*SNORA67*	small nucleolar RNA, H/ACA box 67	26781	17p13.1
26784_at	*SNORA64*	small nucleolar RNA, H/ACA box 64	26784	16p13.3
26828_at	*RNU5F-1*	RNA, U5F small nuclear 1	26828	1p34.1
26832_at	*RNU5B-1*	RNA, U5B small nuclear 1	26832	15q22.31
27074_at	*LAMP3*	lysosomal associated membrane protein 3	27074	3q27.1
27134_at	*TJP3*	tight junction protein 3	27134	19p13.3
27284_at	*SULT1B1*	sulfotransferase family 1B member 1	27284	4q13.3
27285_at	*TEKT2*	tektin 2	27285	1p34.3
27286_at	*SRPX2*	sushi repeat containing protein X-linked 2	27286	Xq22.1
27348_at	*TOR1B*	torsin family 1 member B	27348	9q34.11
28474_at	*IGHV1-2*	immunoglobulin heavy variable 1-2	28474	14q32.33
28567_at	*TRBV20-1*	T cell receptor beta variable 20-1	28567	7q34
28946_at	*IGKJ5*	immunoglobulin kappa joining 5	28946	2p11.2
28972_at	*SPCS1*	signal peptidase complex subunit 1	28972	3p21.1
29015_at	*SLC43A3*	solute carrier family 43 member 3	29015	11q12.1

29108_at	*PYCARD*	PYD and CARD domain containing	29108	16p11.2
29126_at	*CD274*	CD274 molecule	29126	9p24.1
29887_at	*SNX10*	sorting nexin 10	29887	7p15.2
30836_at	*DNTTIP2*	deoxynucleotidyltransferase terminal interacting protein 2	30836	1p22.1
51131_at	*PHF11*	PHD finger protein 11	51131	13q14.2
51177_at	*PLEKHO1*	pleckstrin homology domain containing O1	51177	1q21.2
51191_at	*HERC5*	HECT and RLD domain containing E3 ubiquitin protein ligase 5	51191	4q22.1
51246_at	*SHISA5*	shisa family member 5	51246	3p21.31
51278_at	*IER5*	immediate early response 5	51278	1q25.3
51311_at	*TLR8*	toll like receptor 8	51311	Xp22.2
51439_at	*FAM8A1*	family with sequence similarity 8 member A1	51439	6p22.3
51513_at	*ETV7*	ETS variant transcription factor 7	51513	6p21.31
51604_at	*PIGT*	phosphatidylinositol glycan anchor biosynthesis class T	51604	20q13.12
51646_at	*YPEL5*	yippee like 5	51646	2p23.1
51667_at	*NUB1*	negative regulator of ubiquitin like proteins 1	51667	7q36.1
51673_at	*TPPP3*	tubulin polymerization promoting protein family member 3	51673	16q22.1
51727_at	*CMPK1*	cytidine/uridine monophosphate kinase 1	51727	1p33
51734_at	*MSRB1*	methionine sulfoxide reductase B1	51734	16p13.3
51742_at	*ARID4B*	AT-rich interaction domain 4B	51742	1q42.3
51776_at	*MAP3K20*	mitogen-activated protein kinase kinase kinase 20	51776	2q31.1
51804_at	*SIX4*	SIX homeobox 4	51804	14q23.1
53833_at	*IL20RB*	interleukin 20 receptor subunit beta	53833	3q22.3
53840_at	*TRIM34*	tripartite motif containing 34	53840	11p15.4
53905_at	*DUOX1*	dual oxidase 1	53905	15q21.1
53947_at	*A4GALT*	alpha 1,4-galactosyltransferase (P blood group)	53947	22q13.2
54206_at	*ERRFI1*	ERBB receptor feedback inhibitor 1	54206	1p36.23
54407_at	*SLC38A2*	solute carrier family 38 member 2	54407	12q13.11
54464_at	*XRN1*	5'-3' exoribonuclease 1	54464	3q23
54504_at	*CPVL*	carboxypeptidase vitellogenic like	54504	7p14.3
54509_at	*RHOF*	ras homolog family member F, filopodia associated	54509	12q24.31
54544_at	*CRCT1*	cysteine rich C-terminal 1	54544	1q21.3
54602_at	*NDFIP2*	Nedd4 family interacting protein 2	54602	13q31.1

54625_at	*PARP14*	poly(ADP-ribose) polymerase family member 14	54625	3q21.1
54739_at	*XAF1*	XIAP associated factor 1	54739	17p13.1
54809_at	*SAMD9*	sterile alpha motif domain containing 9	54809	7q21.2
54877_at	*ZCCHC2*	zinc finger CCHC-type containing 2	54877	18q21.33
54899_at	*PXK*	PX domain containing serine/threonine kinase like	54899	3p14.3
54933_at	*RHBDL2*	rhomboid like 2	54933	1p34.3
54947_at	*LPCAT2*	lysophosphatidylcholine acyltransferase 2	54947	16q12.2
55008_at	*HERC6*	HECT and RLD domain containing E3 ubiquitin protein ligase family member 6	55008	4q22.1
55088_at	*CCDC186*	coiled-coil domain containing 186	55088	10q25.3
55281_at	*TMEM140*	transmembrane protein 140	55281	7q33
55303_at	*GIMAP4*	GTPase, IMAP family member 4	55303	7q36.1
55357_at	*TBC1D2*	TBC1 domain family member 2	55357	9q22.33
55454_at	*CSGALNACT2*	chondroitin sulfate N-acetylgalactosaminyltransferase 2	55454	10q11.21
55600_at	*ITLN1*	intelectin 1	55600	1q23.3
55601_at	*DDX60*	DExD/H-box helicase 60	55601	4q32.3
55640_at	*FLVCR2*	feline leukemia virus subgroup C cellular receptor family member 2	55640	14q24.3
55752_at	*SEPTIN11*	septin 11	55752	4q21.1
55824_at	*PAG1*	phosphoprotein membrane anchor with glycosphingolipid microdomains 1	55824	8q21.13
55907_at	*CMAS*	cytidine monophosphate N-acetylneuraminic acid synthetase	55907	12p12.1
56204_at	*FAM214A*	family with sequence similarity 214 member A	56204	15q21.2-q21.3
56664_at	*VTRNA1-1*	vault RNA 1-1	56664	5q31.3
56667_at	*MUC13*	mucin 13, cell surface associated	56667	3q21.2
56683_at	*CFAP298*	cilia and flagella associated protein 298	56683	21q22.11
56829_at	*ZC3HAV1*	zinc finger CCCH-type containing, antiviral 1	56829	7q34
56913_at	*C1GALT1*	core 1 synthase, glycoprotein-N-acetylgalactosamine 3-beta-galactosyltransferase 1	56913	7p22.1-p21.3
57016_at	*AKR1B10*	aldo-keto reductase family 1 member B10	57016	7q33
57091_at	*CASS4*	Cas scaffold protein family member 4	57091	20q13.31
57126_at	*CD177*	CD177 molecule	57126	19q13.31
57402_at	*S100A14*	S100 calcium binding protein A14	57402	1q21.3
57447_at	*NDRG2*	NDRG family member 2	57447	14q11.2

57522_at	*SRGAP1*	SLIT-ROBO Rho GTPase activating protein 1	57522	12q14.2
57568_at	*SIPA1L2*	signal induced proliferation associated 1 like 2	57568	1q42.2
57655_at	*GRAMD1A*	GRAM domain containing 1A	57655	19q13.11
57674_at	*RNF213*	ring finger protein 213	57674	17q25.3
57730_at	*ANKRD36B*	ankyrin repeat domain 36B	57730	2q11.2
58484_at	*NLRC4*	NLR family CARD domain containing 4	58484	2p22.3
58489_at	*ABHD17C*	abhydrolase domain containing 17C	58489	15q25.1
58505_at	*OSTC*	oligosaccharyltransferase complex non-catalytic subunit	58505	4q25
64065_at	*PERP*	p53 apoptosis effector related to PMP22	64065	6q23.3
64073_at	*C19orf33*	chromosome 19 open reading frame 33	64073	19q13.2
64108_at	*RTP4*	receptor transporter protein 4	64108	3q27.3
64135_at	*IFIH1*	interferon induced with helicase C domain 1	64135	2q24.2
64174_at	*DPEP2*	dipeptidase 2	64174	16q22.1
64319_at	*FBRS*	fibrosin	64319	16p11.2
64761_at	*PARP12*	poly(ADP-ribose) polymerase family member 12	64761	7q34
65220_at	*NADK*	NAD kinase	65220	1p36.33
66004_at	*LYNX1*	Ly6/neurotoxin 1	66004	8q24.3
79132_at	*DHX58*	DExH-box helicase 58	79132	17q21.2
79134_at	*TMEM185B*	transmembrane protein 185B	79134	2q14.2
79572_at	*ATP13A3*	ATPase 13A3	79572	3q29
79627_at	*OGFRL1*	opioid growth factor receptor like 1	79627	6q13
79651_at	*RHBDF2*	rhomboid 5 homolog 2	79651	17q25.1
79660_at	*PPP1R3B*	protein phosphatase 1 regulatory subunit 3B	79660	8p23.1
79682_at	*CENPU*	centromere protein U	79682	4q35.1
79838_at	*TMC5*	transmembrane channel like 5	79838	16p12.3
80004_at	*ESRP2*	epithelial splicing regulatory protein 2	80004	16q22.1
80216_at	*ALPK1*	alpha kinase 1	80216	4q25
80231_at	*CXorf21*	chromosome X open reading frame 21	80231	Xp21.2
80380_at	*PDCD1LG2*	programmed cell death 1 ligand 2	80380	9p24.1
80774_at	*LIMD2*	LIM domain containing 2	80774	17q23.3
80830_at	*APOL6*	apolipoprotein L6	80830	22q12.3
81030_at	*ZBP1*	Z-DNA binding protein 1	81030	20q13.31
81031_at	*SLC2A10*	solute carrier family 2 member 10	81031	20q13.12
81610_at	*FAM83D*	family with sequence similarity 83 member D	81610	20q11.23
81876_at	*RAB1B*	RAB1B, member RAS oncogene family	81876	11q13.2

83661_at	*MS4A8*	membrane spanning 4-domains A8	83661	11q12.2
83666_at	*PARP9*	poly(ADP-ribose) polymerase family member 9	83666	3q21.1
83999_at	*KREMEN1*	kringle containing transmembrane protein 1	83999	22q12.1
84034_at	*EMILIN2*	elastin microfibril interfacer 2	84034	18p11.32-p11.31
84134_at	*TOMM40L*	translocase of outer mitochondrial membrane 40 like	84134	1q23.3
84166_at	*NLRC5*	NLR family CARD domain containing 5	84166	16q13
84340_at	*GFM2*	G elongation factor mitochondrial 2	84340	5q13.3
84418_at	*CYSTM1*	cysteine rich transmembrane module containing 1	84418	5q31.3
84419_at	*C15orf48*	chromosome 15 open reading frame 48	84419	15q21.1
84518_at	*CNFN*	cornifelin	84518	19q13.2
84628_at	*NTNG2*	netrin G2	84628	9q34.13
84651_at	*SPINK7*	serine peptidase inhibitor, Kazal type 7 (putative)	84651	5q32
84658_at	*ADGRE3*	adhesion G protein-coupled receptor E3	84658	19p13.12
84868_at	*HAVCR2*	hepatitis A virus cellular receptor 2	84868	5q33.3
84898_at	*PLXDC2*	plexin domain containing 2	84898	10p12.31
84919_at	*PPP1R15B*	protein phosphatase 1 regulatory subunit 15B	84919	1q32.1
84941_at	*HSH2D*	hematopoietic SH2 domain containing	84941	19p13.11
85363_at	*TRIM5*	tripartite motif containing 5	85363	11p15.4
85389_at	*SNORD14C*	small nucleolar RNA, C/D box 14C	85389	11q24.1
85403_at	*EAF1*	ELL associated factor 1	85403	3p25.1
85415_at	*RHPN2*	rhophilin Rho GTPase binding protein 2	85415	19q13.11
85441_at	*HELZ2*	helicase with zinc finger 2	85441	20q13.33
87178_at	*PNPT1*	polyribonucleotide nucleotidyltransferase 1	87178	2p16.1
90861_at	*JPT2*	Jupiter microtubule associated homolog 2	90861	16p13.3
90865_at	*IL33*	interleukin 33	90865	9p24.1
91056_at	*AP5B1*	adaptor related protein complex 5 subunit beta 1	91056	11q13.1
91351_at	*DDX60L*	DExD/H-box 60 like	91351	4q32.3
91543_at	*RSAD2*	radical S-adenosyl methionine domain containing 2	91543	2p25.2
92241_at	*RCSD1*	RCSD domain containing 1	92241	1q24.2
92291_at	*CAPN13*	calpain 13	92291	2p23.1
93432_at	*MGAM2*	maltase-glucoamylase 2 (putative)	93432	7q34
93978_at	*CLEC6A*	C-type lectin domain containing 6A	93978	12p13.31

94163_at	*SNORD38B*	small nucleolar RNA, C/D box 38B	94163	1p34.1
94240_at	*EPSTI1*	epithelial stromal interaction 1	94240	13q14.11
114294_at	*LACTB*	lactamase beta	114294	15q22.2
114599_at	*SNORD15B*	small nucleolar RNA, C/D box 15B	114599	11q13.4
114769_at	*CARD16*	caspase recruitment domain family member 16	114769	11q22.3
114882_at	*OSBPL8*	oxysterol binding protein like 8	114882	12q21.2
114899_at	*C1QTNF3*	C1q and TNF related 3	114899	5p13.2
115004_at	*CGAS*	cyclic GMP-AMP synthase	115004	6q13
116369_at	*SLC26A8*	solute carrier family 26 member 8	116369	6p21.31
116938_at	*SNORD83B*	small nucleolar RNA, C/D box 83B	116938	22q13.1
118788_at	*PIK3AP1*	phosphoinositide-3-kinase adaptor protein 1	118788	10q24.1
118932_at	*ANKRD22*	ankyrin repeat domain 22	118932	10q23.31
120425_at	*JAML*	junction adhesion molecule like	120425	11q23.3
123036_at	*TC2N*	tandem C2 domains, nuclear	123036	14q32.12
124637_at	*CYB5D1*	cytochrome b5 domain containing 1	124637	17p13.1
126820_at	*WDR63*	WD repeat domain 63	126820	1p22.3
128346_at	*C1orf162*	chromosome 1 open reading frame 162	128346	1p13.2
128646_at	*SIRPD*	signal regulatory protein delta	128646	20p13
129607_at	*CMPK2*	cytidine/uridine monophosphate kinase 2	129607	2p25.2
130271_at	*PLEKHH2*	pleckstrin homology, MyTH4 and FERM domain containing H2	130271	2p21
131177_at	*FAM3D*	family with sequence similarity 3 member D	131177	3p14.2
131450_at	*CD200R1*	CD200 receptor 1	131450	3q13.2
132299_at	*OCIAD2*	OCIA domain containing 2	132299	4p11
137886_at	*UBXN2B*	UBX domain protein 2B	137886	8q12.1
145474_at	*LOC145474*	uncharacterized LOC145474	145474	14q24.2
147015_at	*DHRS13*	dehydrogenase/reductase 13	147015	17q11.2
148808_at	*MFSD4A*	major facilitator superfamily domain containing 4A	148808	1q32.1
151636_at	*DTX3L*	deltex E3 ubiquitin ligase 3L	151636	3q21.1
151651_at	*EFHB*	EF-hand domain family member B	151651	3p24.3
151987_at	*PPP4R2*	protein phosphatase 4 regulatory subunit 2	151987	3p13
153222_at	*CREBRF*	CREB3 regulatory factor	153222	5q35.1
154664_at	*ABCA13*	ATP binding cassette subfamily A member 13	154664	7p12.3
162466_at	*PHOSPHO1*	phosphoethanolamine/phosphocholine phosphatase 1	162466	17q21.32
162517_at	*FBXO39*	F-box protein 39	162517	17p13.1
163259_at	*DENND2C*	DENN domain containing 2C	163259	1p13.2

193629_at	*LINC00189*	long intergenic non-protein coding RNA 189	193629	21q21.3
197259_at	*MLKL*	mixed lineage kinase domain like pseudokinase	197259	16q23.1
199675_at	*MCEMP1*	mast cell expressed membrane protein 1	199675	19p13.2
200931_at	*SLC51A*	solute carrier family 51 alpha subunit	200931	3q29
219285_at	*SAMD9L*	sterile alpha motif domain containing 9 like	219285	7q21.2
219972_at	*MPEG1*	macrophage expressed 1	219972	11q12.1
219988_at	*PATL1*	PAT1 homolog 1, processing body mRNA decay factor	219988	11q12.1
220929_at	*ZNF438*	zinc finger protein 438	220929	10p11.23
221037_at	*JMJD1C*	jumonji domain containing 1C	221037	10q21.3
222487_at	*ADGRG3*	adhesion G protein-coupled receptor G3	222487	16q21
245973_at	*ATP6V1C2*	ATPase H+ transporting V1 subunit C2	245973	2p25.1
255488_at	*RNF144B*	ring finger protein 144B	255488	6p22.3
256144_at	*OR4C3*	olfactory receptor family 4 subfamily C member 3	256144	11p11.2
267010_at	*RNU12*	RNA, U12 small nuclear	267010	22q13.2
284207_at	*METRNL*	meteorin like, glial cell differentiation regulator	284207	17q25.3
284454_at	*LOC284454*	uncharacterized LOC284454	284454	19p13.12
285855_at	*RPL7L1*	ribosomal protein L7 like 1	285855	6p21.1
286002_at	*SLC26A4-AS1*	SLC26A4 antisense RNA 1	286002	7q22.3
317772_at	*H2AC21*	H2A clustered histone 21	317772	1q21.2
319103_at	*SNORD8*	small nucleolar RNA, C/D box 8	319103	14q11.2
337873_at	*H2BC20P*	H2B clustered histone 20, pseudogene	337873	1q21.2
338339_at	*CLEC4D*	C-type lectin domain family 4 member D	338339	12p13.31
338758_at	*ATP2B1-AS1*	ATP2B1 antisense RNA 1	338758	12q21.33
340198_at	*IFITM4P*	interferon induced transmembrane protein 4 pseudogene	340198	6p22.1
343070_at	*PRAMEF9*	PRAME family member 9	343070	1p36.21
345930_at	*ECT2L*	epithelial cell transforming 2 like	345930	6q24.1
353135_at	*LCE1E*	late cornified envelope 1E	353135	1q21.3
353345_at	*GPR141*	G protein-coupled receptor 141	353345	7p14.1
353514_at	*LILRA5*	leukocyte immunoglobulin like receptor A5	353514	19q13.42
359845_at	*RFLNB*	refilin B	359845	17p13.3
373156_at	*GSTK1*	glutathione S-transferase kappa 1	373156	7q34
377677_at	*CA13*	carbonic anhydrase 13	377677	8q21.2
378108_at	*TRIM74*	tripartite motif containing 74	378108	7q11.23
387882_at	*C12orf75*	chromosome 12 open reading frame 75	387882	12q23.3

388633_at	*LDLRAD1*	low density lipoprotein receptor class A domain containing 1	388633	1p32.3
391257_at	*SUMO1P1*	SUMO1 pseudogene 1	391257	20q13.2
399665_at	*FAM102A*	family with sequence similarity 102 member A	399665	9q34.11
399948_at	*COLCA1*	colorectal cancer associated 1	399948	11q23.1
399949_at	*C11orf88*	chromosome 11 open reading frame 88	399949	11q23.1
399972_at	*GSEC*	G-quadruplex forming sequence containing lncRNA	399972	11q24.2
400499_at	*LOC400499*	putative uncharacterized protein LOC400499	400499	16p13.13
400759_at	*GBP1P1*	guanylate binding protein 1 pseudogene 1	400759	1p22.2
401551_at	*WDR38*	WD repeat domain 38	401551	9q33.3
407018_at	*MIR27A*	microRNA 27a	407018	19p13.12
440742_at	*LOC440742*	uncharacterized LOC440742	440742	1q44
441168_at	*CALHM6*	calcium homeostasis modulator family member 6	441168	6q22.1
574406_at	*ADAMTSL4-AS1*	ADAMTSL4 antisense RNA 1	574406	1q21.2
574484_at	*MIR520G*	microRNA 520g	574484	19q13.42
594839_at	*SNORA33*	small nucleolar RNA, H/ACA box 33	594839	6q23.2
619505_at	*SNORA21*	small nucleolar RNA, H/ACA box 21	619505	17q12
619569_at	*SNORA41*	small nucleolar RNA, H/ACA box 41	619569	2q33.3
643161_at	*FAM25A*	family with sequence similarity 25 member A	643161	10q23.2
643802_at	*LOC643802*	u3 small nucleolar ribonucleoprotein protein MPP10-like	643802	16q12.2
647309_at	*GMNC*	geminin coiled-coil domain containing	647309	3q28
654321_at	*SNORA75*	small nucleolar RNA, H/ACA box 75	654321	2q37.1
677679_at	*SCARNA3*	small Cajal body-specific RNA 3	677679	1q25.1
677767_at	*SCARNA7*	small Cajal body-specific RNA 7	677767	3q25.33
677770_at	*SCARNA22*	small Cajal body-specific RNA 22	677770	4p16.3
677771_at	*SCARNA4*	small Cajal body-specific RNA 4	677771	1q22
677772_at	*SCARNA6*	small Cajal body-specific RNA 6	677772	2q37.1
677776_at	*SCARNA8*	small Cajal body-specific RNA 8	677776	9p22.1
677781_at	*SCARNA16*	small Cajal body-specific RNA 16	677781	17q25.2
677793_at	*SNORA2A*	small nucleolar RNA, H/ACA box 2A	677793	12q13.11
677794_at	*SNORA2B*	small nucleolar RNA, H/ACA box 2B	677794	12q13.11
677798_at	*SNORA9*	small nucleolar RNA, H/ACA box 9	677798	7p13
677800_at	*SNORA12*	small nucleolar RNA, H/ACA box 12	677800	10q24.31
677801_at	*SNORA14A*	small nucleolar RNA, H/ACA box 14A	677801	7q11.23
677805_at	*SNORA18*	small nucleolar RNA, H/ACA box 18	677805	11q21
677806_at	*SNORA20*	small nucleolar RNA, H/ACA box 20	677806	6q25.3

677808_at	*SNORA23*	small nucleolar RNA, H/ACA box 23	677808	11p15.4
677809_at	*SNORA24*	small nucleolar RNA, H/ACA box 24	677809	4q26
677810_at	*SNORA26*	small nucleolar RNA, H/ACA box 26	677810	4q12
677815_at	*SNORA2C*	small nucleolar RNA, H/ACA box 2C	677815	12q13.11
677819_at	*SNORA37*	small nucleolar RNA, H/ACA box 37	677819	18q21.2
677826_at	*SNORA3B*	small nucleolar RNA, H/ACA box 3B	677826	11p15.4
677829_at	*SNORA49*	small nucleolar RNA, H/ACA box 49	677829	12q24.33
677830_at	*SNORA50A*	small nucleolar RNA, H/ACA box 50A	677830	16q21
677837_at	*SNORA60*	small nucleolar RNA, H/ACA box 60	677837	20q11.23
677838_at	*SNORA61*	small nucleolar RNA, H/ACA box 61	677838	1p35.3
677839_at	*SNORA71C*	small nucleolar RNA, H/ACA box 71C	677839	20q11.23
677842_at	*SNORA50C*	small nucleolar RNA, H/ACA box 50C	677842	17q23.3
692053_at	*SNORD9*	small nucleolar RNA, C/D box 9	692053	14q11.2
692148_at	*SCARNA10*	small Cajal body-specific RNA 10	692148	12p13.31
692149_at	*SCARNA14*	small Cajal body-specific RNA 14	692149	15q22.31
693199_at	*MIR614*	microRNA 614	693199	12p13.1
730249_at	*ACOD1*	aconitate decarboxylase 1	730249	13q22.3
100126299_at	*VTRNA2-1*	vault RNA 2-1	100126299	5q31.1
100128731_at	*OST4*	oligosaccharyltransferase complex subunit 4, non-catalytic	100128731	2p23.3
100130733_at	*LRRC70*	leucine rich repeat containing 70	100130733	5q12.1
100151683_at	*RNU4ATAC*	RNA, U4atac small nuclear (U12-dependent splicing)	100151683	2q14.2
100271836_at	*SMG1P3*	SMG1 pseudogene 3	100271836	16p12.2
100289462_at	*DEFB4B*	defensin beta 4B	100289462	8p23.1
100506229_at	*LINC01093*	long intergenic non-protein coding RNA 1093	100506229	4q35.1
100616282_at	*MIR4738*	microRNA 4738	100616282	17q25.1
101927015_at	*LINC01506*	long intergenic non-protein coding RNA 1506	101927015	9q21.11
101927017_at	*LINC01180*	long intergenic non-protein coding RNA 1180	101927017	17q21.31
101927153_at	*LINC02207*	long intergenic non-protein coding RNA 2207	101927153	15q26.2
101929207_at	*LINC01729*	long intergenic non-protein coding RNA 1729	101929207	20p12.3
102724515_at	*LINC01291*	long intergenic non-protein coding RNA 1291	102724515	2p12

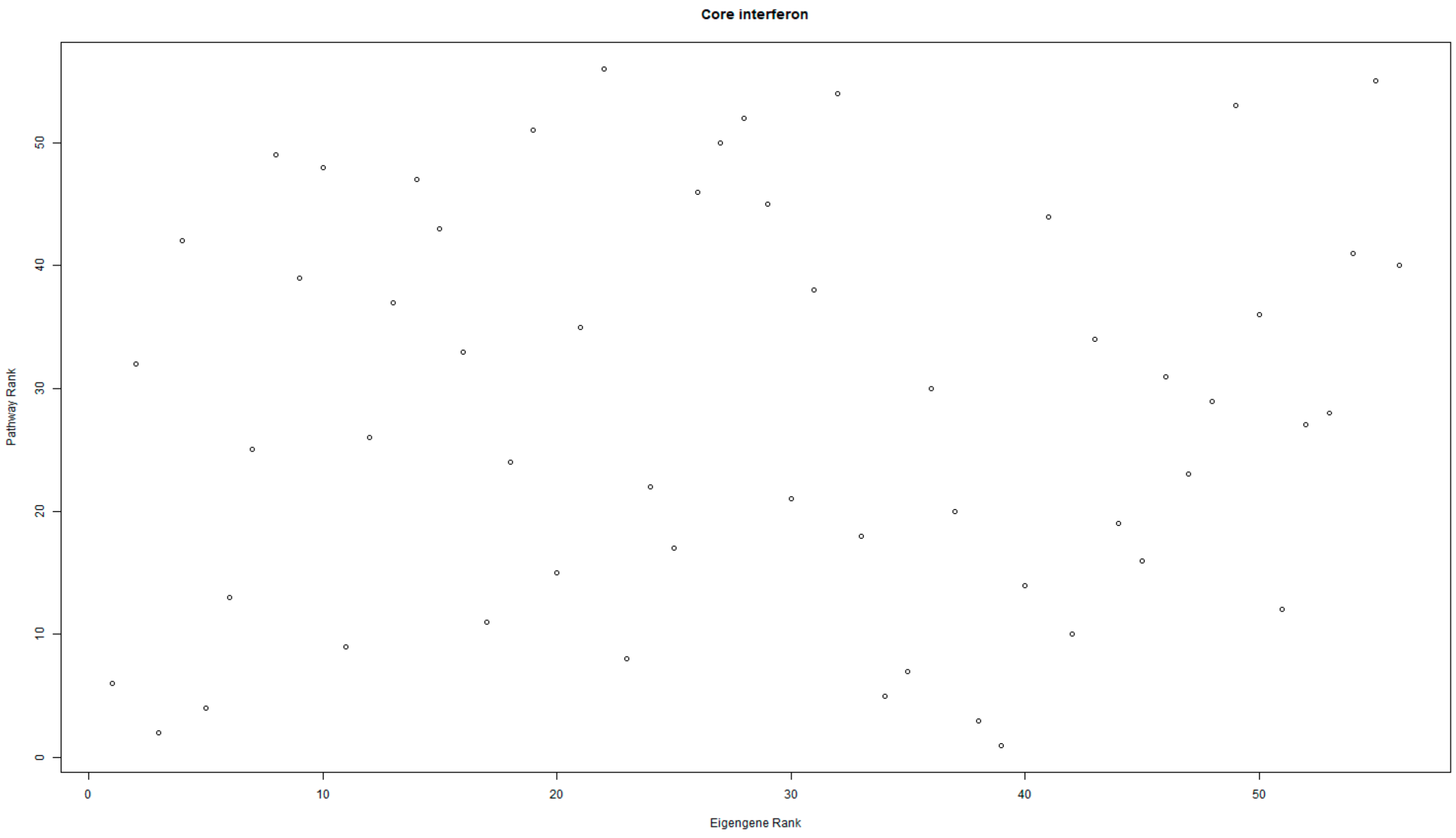

Figure S1. Individual ranks for signature eigengenes versus core interferon pathway proportions.

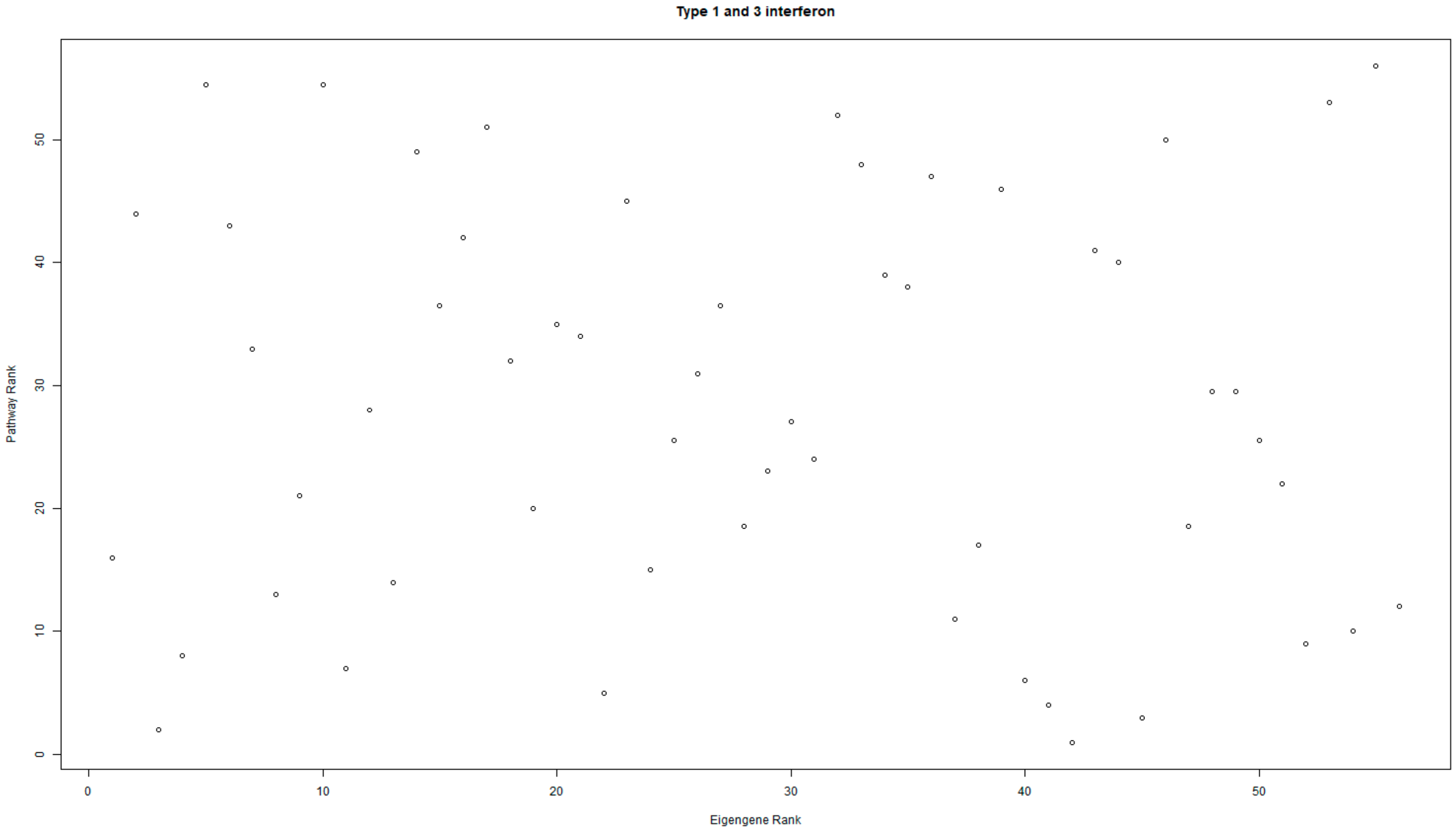

Figure S2. Individual ranks for signature eigengenes versus type 1 and 3 interferon pathway proportions.

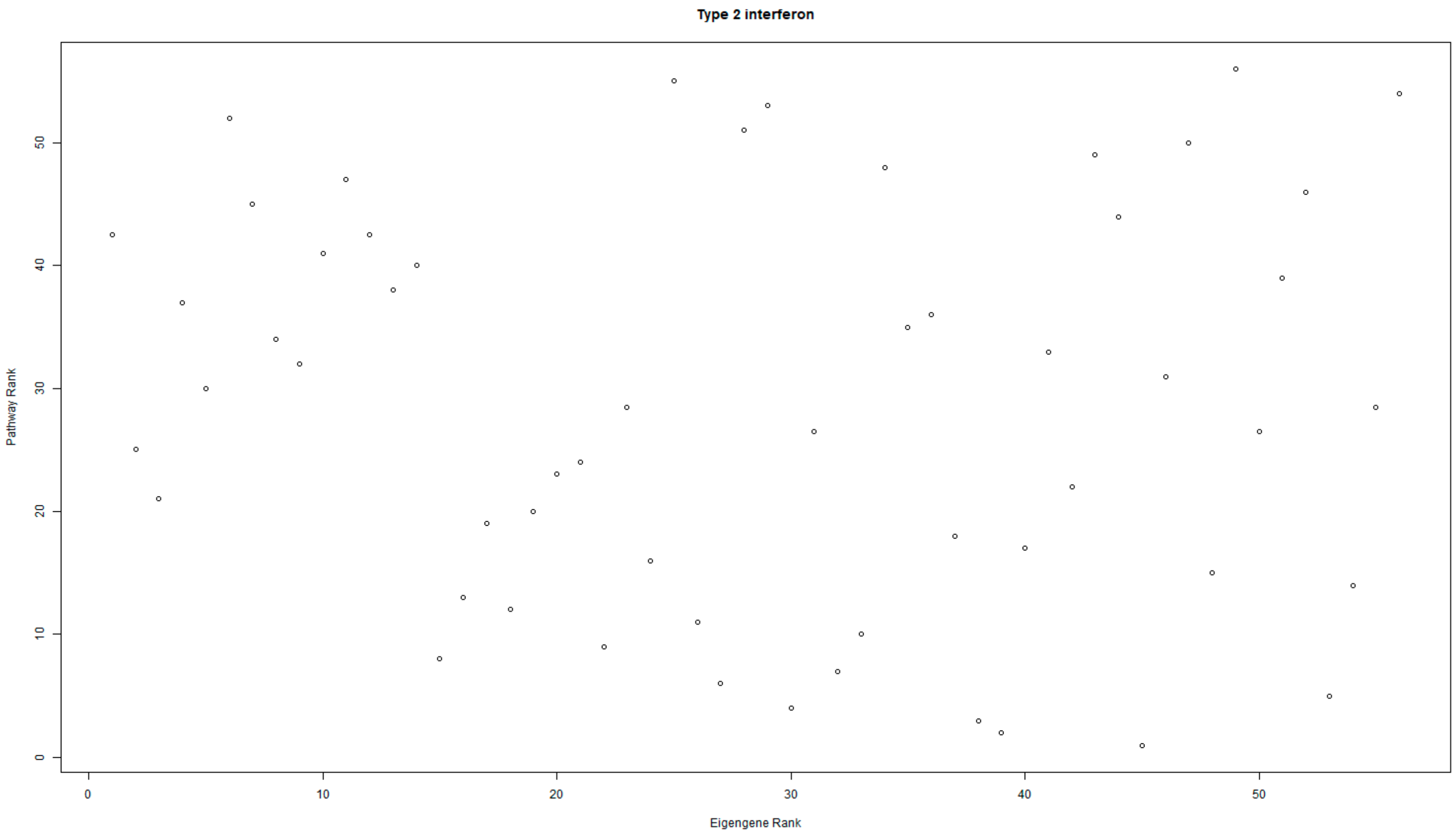

Figure S3. Individual ranks for signature eigengenes versus type 2 interferon pathway proportions.

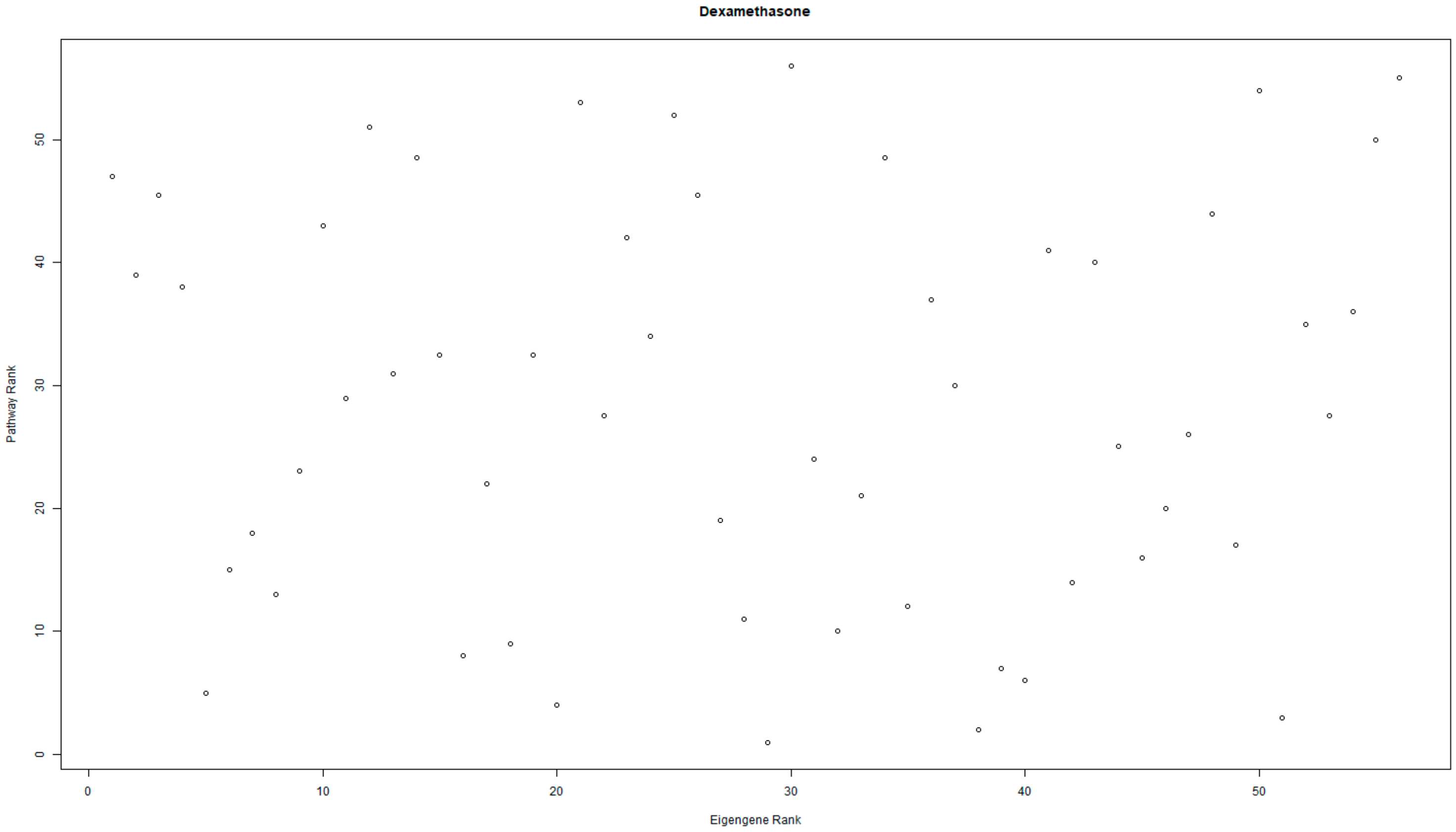

Figure S4. Individual ranks for signature eigengenes versus dexamethasone pathway proportions.

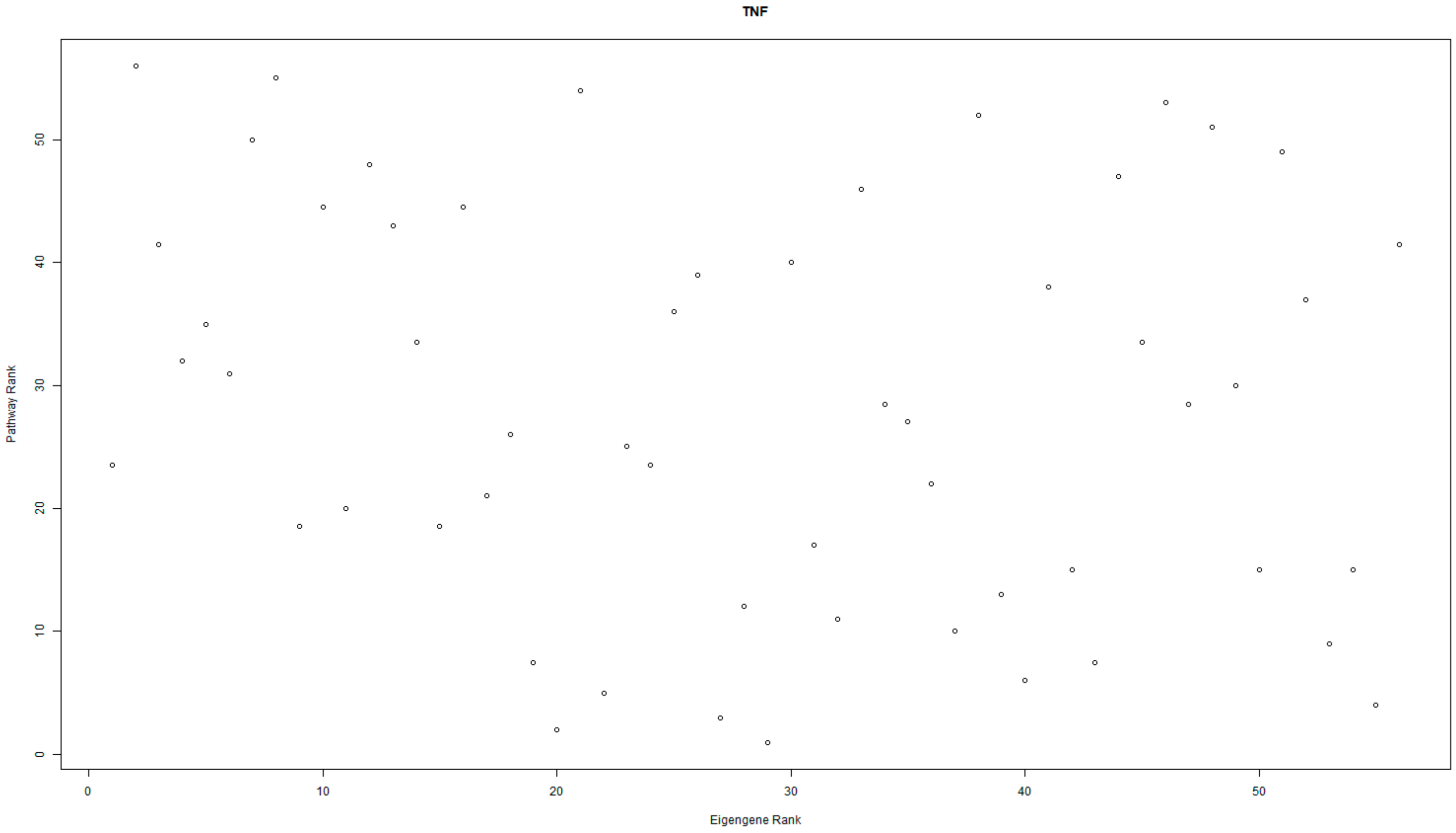

Figure S5. Individual ranks for signature eigengenes versus TNF pathway proportions.

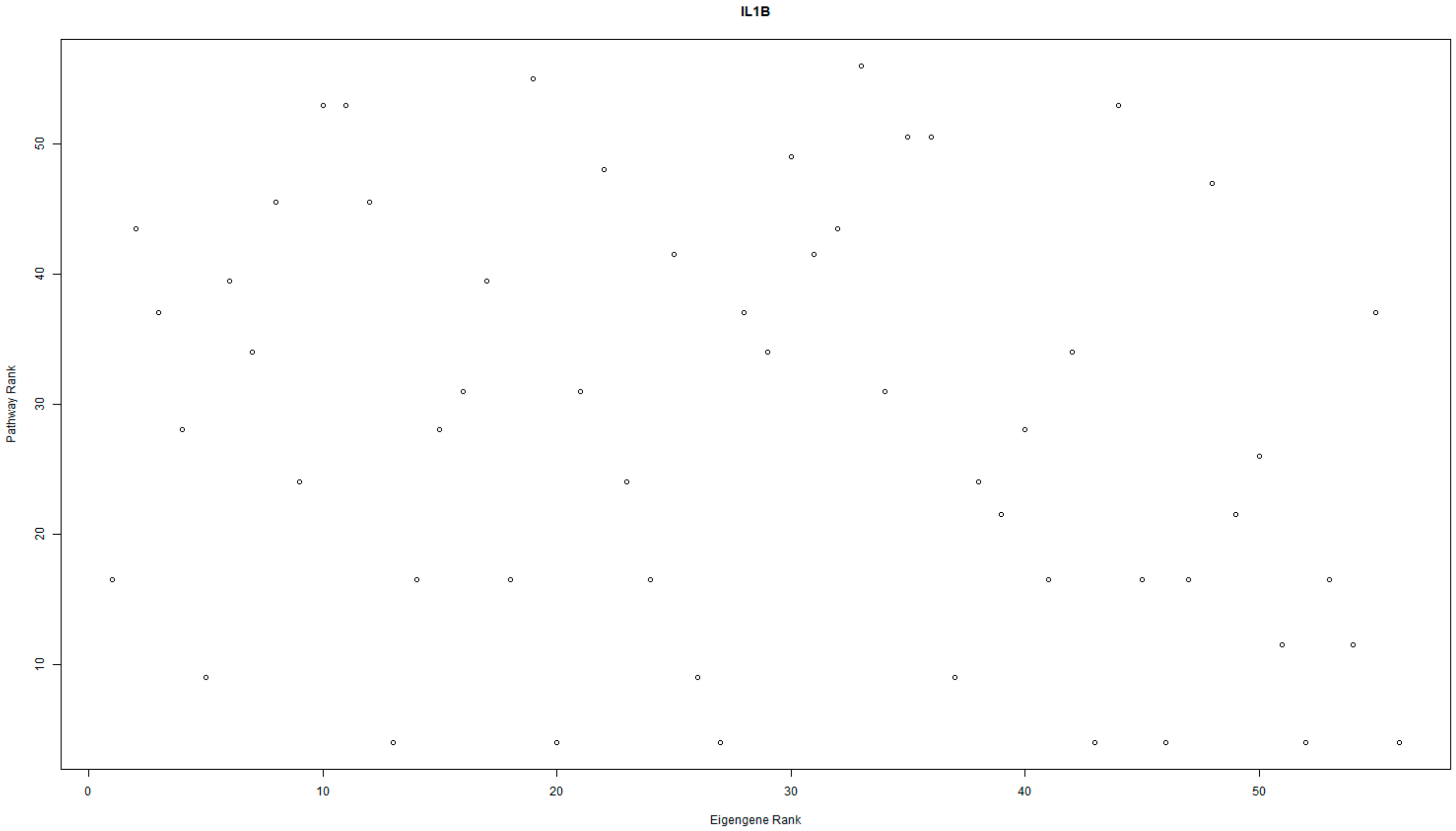

Figure S6. Individual ranks for signature eigengenes versus IL1B pathway proportions.

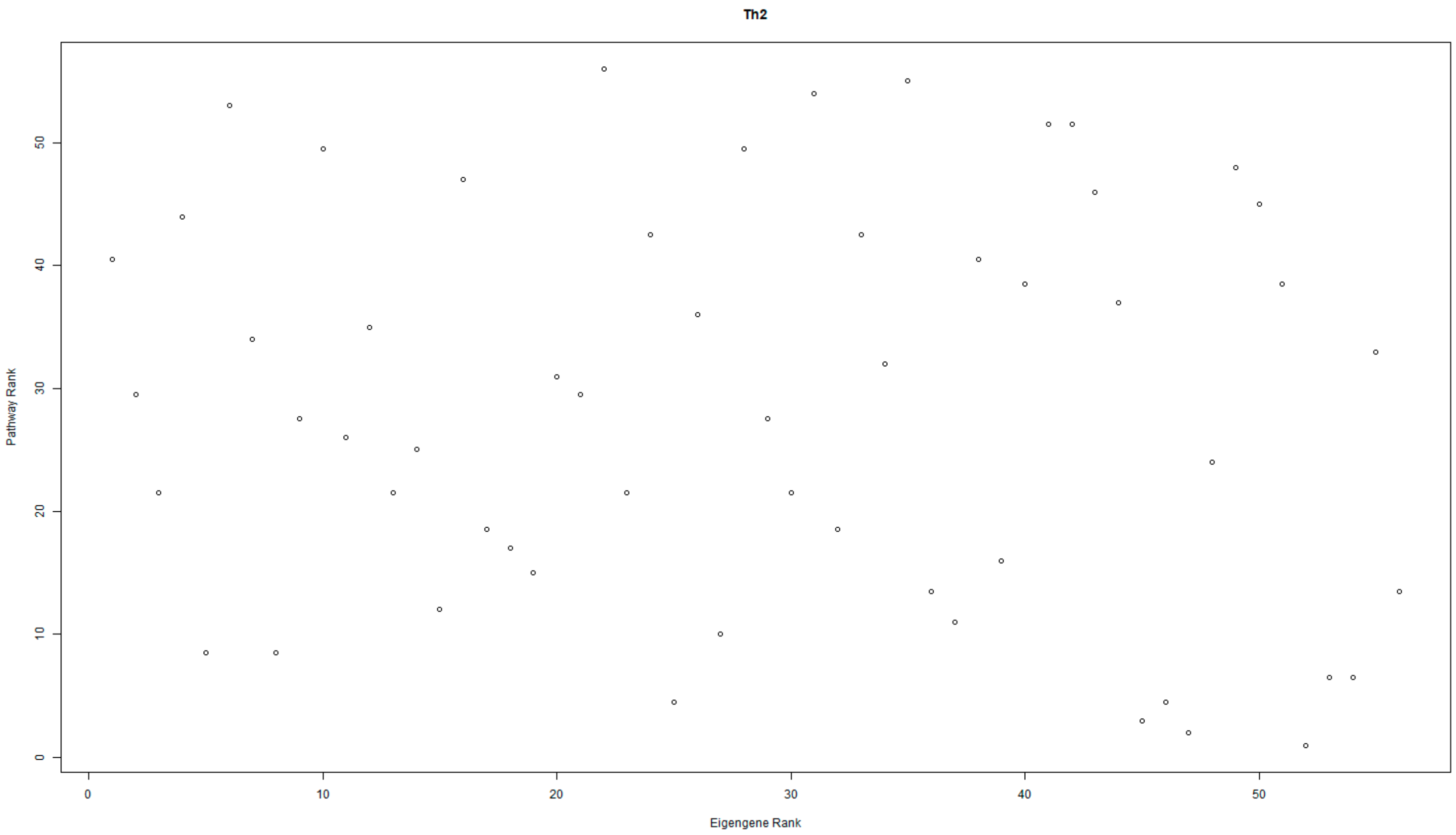

Figure S7. Individual ranks for signature eigengenes versus Th2 pathway proportions.

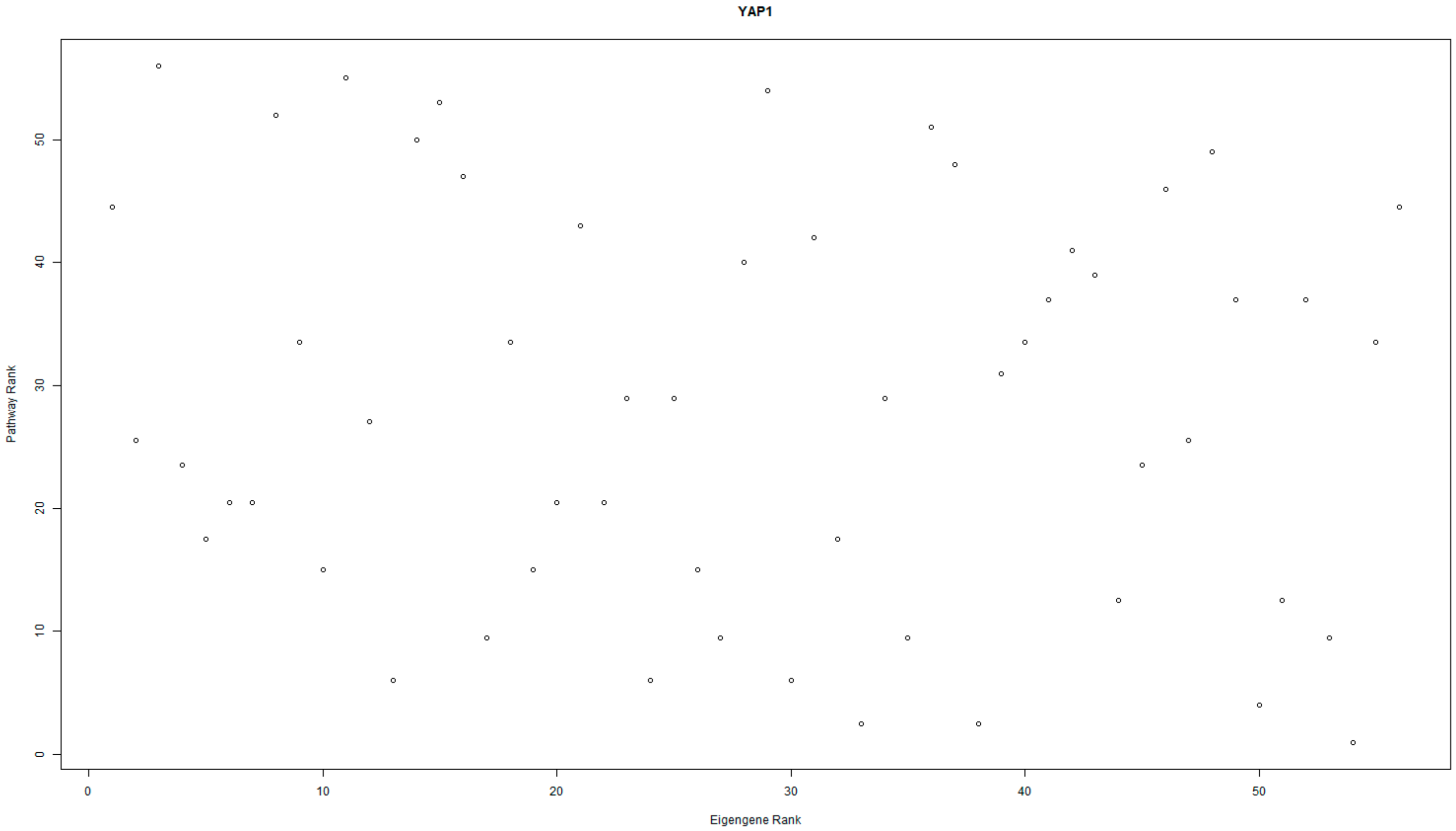

Figure S8. Individual ranks for signature eigengenes versus YAP1 pathway proportions.

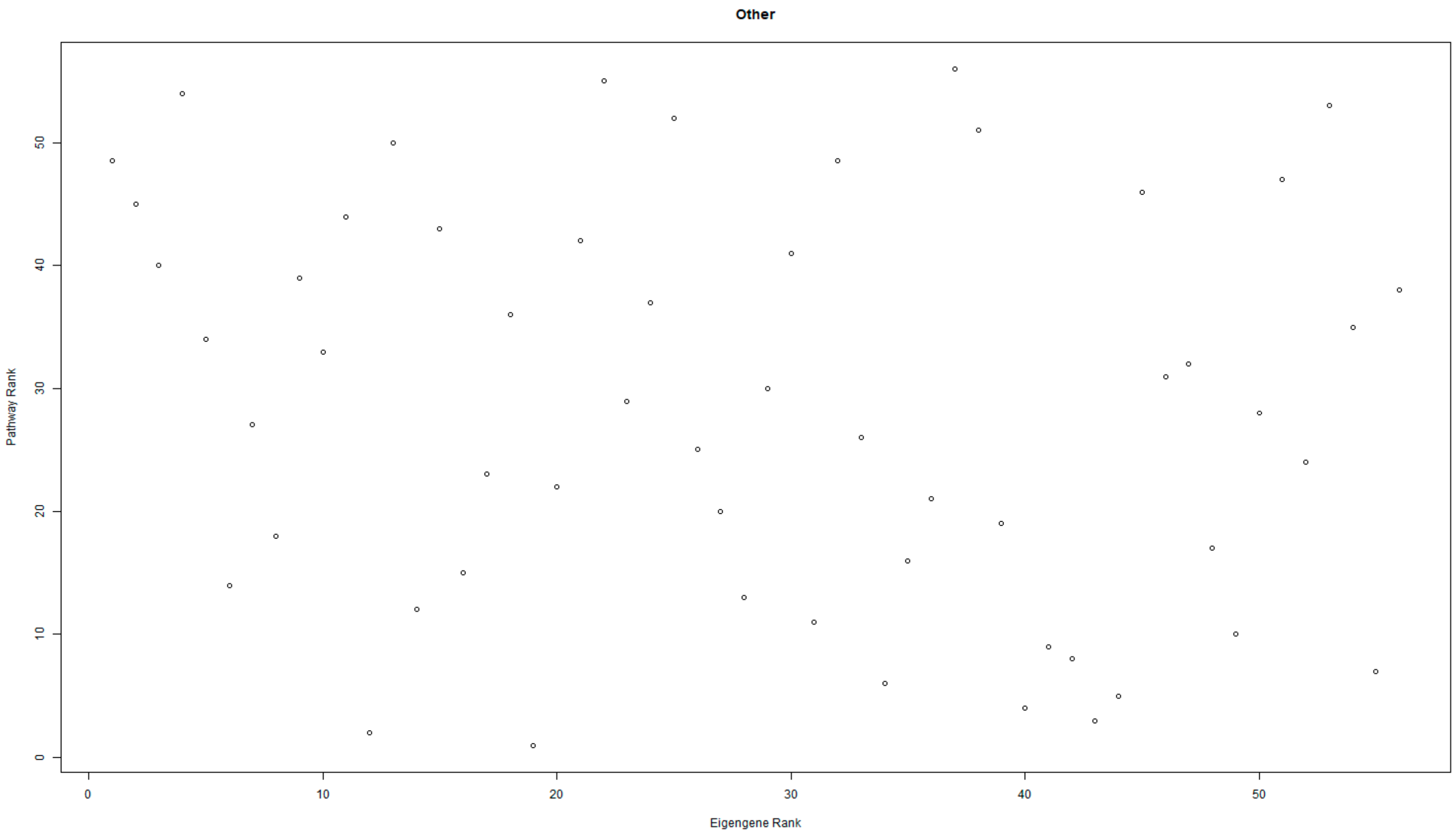

Figure S9. Individual ranks for signature eigengenes versus "other" pathway proportions.
